# Currently managed US prevalence of cutaneous venous malformations (cVMs): a nationally representative, retrospective, real-world, subject-blinded, physician-observational probability study

**DOI:** 10.1186/s13023-025-03995-8

**Published:** 2025-10-07

**Authors:** Jack Ray Gallagher, Susan Carroll, Jeffrey Martini, Michael Kelly, Maria Gnarra Buethe

**Affiliations:** 1 Clarity Pharma Research, LLC, 511 Audubon, Spartanburg, SC 29302 USA; 2 Palvella Therapeutics, Inc, 125 Strafford Ave., Suite 360, Wayne, PA 19087 USA; 3 Department of Pediatric Hematology Oncology and Blood Marrow Transplantation, Cleveland Clinic, 8950 Euclid Ave, Cleveland, OH 44195 USA; 4 LGDA, 7901 4th St. North Suite 5761, St. Petersburg, FL 33702 USA; 5 Dermatology Division, Children’s Hospital of Orange Countys / Pediatric Dermatology, University of California Irvine, 1201 W. La Veta Ave., Orange, CA 92868 USA

**Keywords:** Venous malformations, Vascular malformations, Vascular anomalies, Cutaneous venous malformations, Mixed venous malformations, Combined venous malformations, Observational study, Prevalence, Epidemiology

## Abstract

**Background:**

Cutaneous venous malformations (cVMs) are rare vascular anomalies characterized by progressive vessel ectasia, leading to disfigurement, pain, ulceration, and bleeding. These lesions often evade early detection and are notoriously difficult to treat due to the limited dermal bioavailability of systemic therapies. Unlike deep venous malformations, cVMs present unique management challenges and currently lack FDA-approved treatments. To address the paucity of epidemiologic data, we conducted a nationally representative, blinded, real-world observational probability study to estimate the annual treatment prevalence of cVM in the United States.

A geographically representative sample of dermatologists, hematologist-oncologists, pediatricians, radiologists, and vascular surgeons was recruited via blinded invitations. Participants self-reported the number of cVM patients treated in the prior 12 months. Estimates were adjusted for comanagement to calculate the national prevalence of unique patients.

**Results:**

Of 691 physicians who accessed the study website, 515 (74.5%) completed the survey; 376 (73.0%) reported managing at least one patient with cutaneous-only or mixed (cutaneous and internal) VMs. The estimated annual prevalence was 194,195 patients (95% CI 188,852–200,228), including 135,687 with cutaneous-only VMs and 58,508 with mixed lesions. This corresponds to a prevalence of 0.06% (US population).

**Conclusions:**

While cVM is rare, it affects a substantial number of individuals across age groups in the U.S. These findings underscore the need for improved access to care and the development of targeted therapies for this understudied, debilitating condition.

## Background

Venous malformations (VMs) are congenital vascular anomalies arising from abnormal venous development and progressive vessel dilation [1]. VMs can be isolated, regional, or associated with overgrowth syndromes [2]. These lesions may involve cutaneous, subcutaneous, and mucosal tissues, with a predilection for the head, neck, and extremities [3–9]. Though often present at birth, many VMs remain undetected until later in life as symptoms manifest due to vessel ectasia and venous congestion. Clinical manifestations vary by lesion depth, with cutaneous VMs (cVMs) causing ulceration, bleeding, pain, and functional impairment. Management is challenging due to poor dermal penetration of systemic therapies and the lack of FDA-approved pharmacologic options.

Unlike deep VMs, cVMs lack well-established treatment pathways. VM symptoms and therapies are based on the physiological location (cutaneous vs. internal) and include compression, anticoagulation, sclerotherapy, laser therapy, and surgery [3, 4, 10]. Such treatments are not without risk. Sclerotherapy can have complications as well as patient dissatisfaction, and laser therapy can carry the risk of injury or deformation [4, 11–13].

Most cVMs are caused by somatic mutations in TEK or PIK3CA, leading to PI3K/mTOR pathway hyperactivation [3, 4, 14]. Preliminary studies suggest targeted therapies such as sirolimus (mTOR inhibitor) may offer benefit; however, rigorous epidemiologic data to support clinical development and resource allocation remain scarce [15, 16]. Unlike in deep, internal venous malformations, which are located below the skin and are accessible for oral therapies and procedural interventions, cVMs are more difficult to treat because of a lack of accessible targets and limited biodistribution of oral therapies to the dermis [17]. To address the cutaneous component, a targeted topical therapy is needed to address this unmet need.

Previous epidemiologic estimates of vascular anomalies (VAs) are limited, often focused on pediatric incidence or derived from small cohorts. Reported incidence ranges for VMs vary widely (e.g., 1:5000–1:10,000 births) [7], and cVM-specific data are virtually nonexistent [18, 19].Moreover, such estimates fail to capture the full spectrum of patients living with and seeking care for this lifelong disease.

To address these gaps, we conducted the first U.S. prevalence study specifically targeting cVM, including both pediatric and adult patients. Using a validated methodology previously employed in our study of microcystic lymphatic malformations [20],we estimated the national treatment prevalence of cVMs as managed by key specialty providers.

## Methods

We conducted a blinded, nationally representative, retrospective, real-world observational probability study to estimate the annual treatment prevalence of cutaneous venous malformations (cVMs) in the United States. This probability sample methodology was employed to ensure that the survey sample on which the study population prevalence estimations were based would be representative of the target population. This method employs random selection, so that every individual has an equal opportunity to be chosen and to help reduce selection bias [21].

### Study design and sampling

From February 23 to March 12, 2024, we recruited a random probability sample of a group of target specialists that was nationally representative by US Census region, with geographic distributions based on data sources including the American Medical Association (AMA), American Association of Medical Colleges, US Census Bureau, and other verified data sources [22–27].

Our goal was to survey at least 375 physicians who had treated ≥ 1 patient with cutaneous-only or mixed (cutaneous plus internal) VMs within the 12 months preceding participation in the survey. Eligible specialties included dermatologists, hematologists/oncologists, pediatricians, radiologists (interventional, vascular, and diagnostic), and vascular surgeons. Physicians were randomly recruited via email from a national master file of verified patient-care target specialists based on the AMA Masterfile. Physicians received invitations to participate in an online study of a condition that would be disclosed at the study website. The condition was undisclosed at that point to reduce the bias of self-selecting either into or out of the study based on the condition. Moreover, neither the study sponsor nor the respondent knew the other’s identity, an approach used to further reduce the chance of bias.

Important to ensuring representativeness are the confidence level and margin of error. Findings from a representative sample can be generalized to a larger population with a known degree of confidence [28]. For a sample size of 376 physicians out of a target population of 113,766 physicians, one can expect a margin of error of roughly 5% at a 95% confidence level, and for a sample size of 515, the margin of error would be slightly lower, around 4.3% [29]. A 95% confidence level means that if one were to repeat a survey 100 times, 95% of the time the results would fall within the margin of error [30].

When selecting a sample size, one also must consider time, budget, and resources, all of which were factors in designing and conducting this study. We were limited in scope and budget from recruiting all possible specialties that might be part of a particular cVM patient’s team, but we asked survey participants to identify the other types of specialists who were on their cVM patients’ comanagement teams and factored those responses into our estimations [31–33].

### Survey procedures

Participants confirmed their specialty, practice type, and annual patient volume. They were then shown standardized clinical images and descriptions of cVM. Respondents indicated whether they had managed any VM patients and, if so, estimated the number with:

- Cutaneous-only VMs
- Internal/deep VMs
- Mixed (cutaneous + internal) VMs

Physicians with cVM experience provided further data on comanagement patterns, age distribution, and specialty overlap.

### Weighting and statistical analysis

To prevent duplication in prevalence estimates, we adjusted responses for comanagement across specialties. Patients reported as managed by multiple providers were assigned fractional weights (e.g., 0.25 if managed by 4 specialists). Outliers in patient volume were identified using SPSS statistical tools (e.g., boxplots, quartiles, histograms) and replaced with median values stratified by practice setting (vascular anomaly center vs. community/private practice).

Statistical analyses included t-tests and ANOVA for continuous variables, and chi-square or z-tests for categorical comparisons, with Bonferroni correction for multiple comparisons. All results were evaluated at a 0.05 significance threshold.

## Results

Among 691 invited physicians who accessed the survey, 515 (74.5%) completed it. Of these, 401 (77.9%) had treated at least one VM patient, and 376 (73.0%) had managed at least one patient with a cutaneous-only or mixed VM.

### National prevalence estimates

The model estimated an annual total of 194,195 (95% CI 188,852–200,228) unique patients treated for cVM:

- Cutaneous-only VMs**:** 135,687 patients (0.04% prevalence rate)
- Mixed VMs: 58,508 patients (0.02% prevalence rate)

**Fig. 1:**
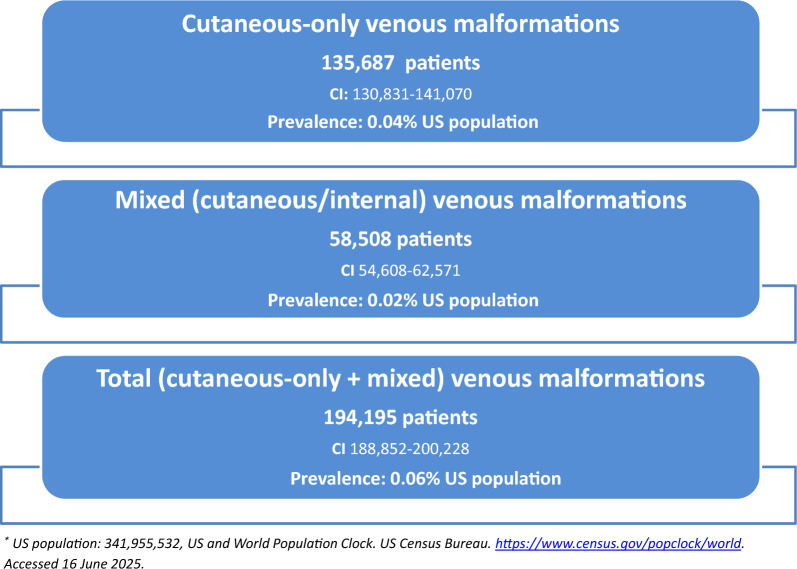
Estimated number of patients treated annually in the US for venous malformations with a cutaneous component

This yields a combined annual prevalence of 0.06% in the 2025 U.S. population (341,955,532) (Table 1, Fig. 1) [34–38].

**Table 1:** Estimates of the number of annual cVM-treated patients in the U.S

	A	B	C	D	E	F	G	H	I
Specialty	MDs in study n = 515	Patient-care target specialists in US n = 113,766	Mean cutaneous- only VM patients/MD	Mean mixed VM (cutaneous/ internal) patients/MD	Mean cutaneous-only PLUS mixed VM patients/MD	Mean unique total patients (any disease) annually/MD	Cutaneous- only VM patients	Mixed VM patients	Total cVM patients
							B*C	B*D	G + H
Pediatrics^1^ [P]	154	54,302	0.88	0.24	1.12	1227	47,786	13,032	60,818
Hematology/ oncology^1^ [H]	119	15,997	1.42	0.90	2.32	850	22,716	14,397	37,113
				P, DM	P				
Dermatology^1^ [DM]	116	12,201	1.39	0.43	1.82	1422	16,959	5246	22,205
			P						
Radiology (vascular and interventional or diagnostic)^1^ [R]	79	27,634	1.41	0.76	2.17	1025	38,964	21,002	59,966
				P	P				
Vascular surgery^1^ [V]	47	3632	2.55	1.33	3.88	1106	9262	4831	14,093
			P,H,DM,R	P,H,DM,R	P,H,DM,R				
Totals*							135,687	58,508	194,195

^*^Co-managed adjusted (weighted) patients; sums and means are subject to rounding 95% CI 130,831–141,070 54,608–62,571 188,852–200,228

^**^For each significant pair, the key (CAPITAL LETTERS P,H,DM,R,V) of the smaller category appears within the category with the larger proportion. Significance level: .05

^1^2022 Physician Specialty Data Report. Association of American Medical Colleges. https://www.aamc.org/data-reports/workforce/data/active-physicians-largest-specialties-major-professional-activity-2021. Accessed 19 Mar 2024

^2^US population of 341,955,532, US and World Population Clock. US Census Bureau. https://www.census.gov/popclock/world. Accessed 16 June 2025. or-professional-activity-2021. Accessed 19 Mar 2024

### Specialty-specific findings

Vascular surgeons reported the highest cVM caseloads (mean 3.88 patients/year), significantly more than dermatologists (1.82), hematologists-oncologists (2.32), pediatricians (1.12), and radiologists (2.17) (*p* < 0.001 for all). Pediatricians were least likely to report managing cutaneous-only or mixed VMs compared with other specialties (*p* < 0.005 across comparisons).

### Geographic distributions

Based on each specialty’s mean number of patients per region:

- Northeast: 53,986 patients
- South: 52,238
- West: 49,326
- Midwest: 38,645

**Fig. 2:**
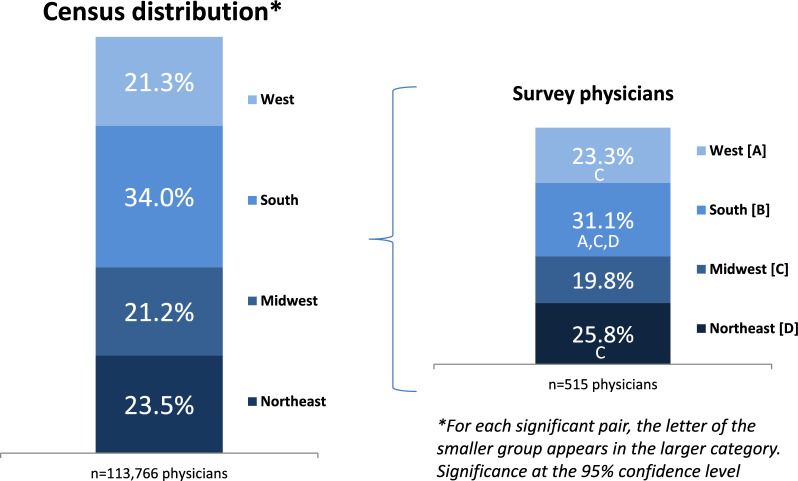
Physician regional distribution. Universe of patient-care pediatricians, dermatologists, diagnostic radiologists, hematologists/oncologists, interventional and vascular radiologists, and vascular surgeons by US Census region. The smaller bar chart at right shows that the regional distribution of specialists participating in the survey closely aligns with the national distribution of these specialists.*Universe regions are an average of specialty regions

Significant differences in cVM patient volume were observed across regions (*p* < 0.001), with the Northeast showing the highest burden (Table 2), although the South had the highest proportion of survey respondents, in keeping with the reported regional distributions of those specialties by U.S. Census region (Fig. 2). In comparing the 376 survey respondents who had cVM patients with the 139 physicians who did not, physicians in the South also were significantly less likely to have cVM patients (*p* = 0.021) (Table 3).

**Table 2:** Estimates of the number of annual cVM-treated patients by region

Estimated number of cVM patients by US Census region^*,**^					
	A	B	C	D	
Region	Northeast	South	West	Midwest	Total
n =	53,986 [BCD]	52,238 [BD]	49,326 [B]	38,645	194,195

^*^Weighted (co-managed adjusted) patients; figures based on each specialty’s mean number of patients per region

^**^For each significant pair, the key (CAPITAL LETTERS A,B,C,D) of the smaller category appears within the category with the larger proportion. Significance level: .05

**Table 3:** Physician characteristics and comparison of those with and without cVM patients

	With cVM patients [A] n = 376 physicians		No cVM patients [B] n = 139 physicians		*p* value	All study physicians n = 515	
Specialty	n	%	n	%	≤ 0.05 bolded	n	%
Pediatricians	96	25.5%	58	41.7% [A]	**.0004**	154	29.9%
Radiologists (vascular & interventional or diagnostic)	50	13.3%	29	20.9% [A]	**.034**	79	15.3%
Hem/Oncs (adult and pediatric)	95	25.3%	24	17.3%	.056	119	23.1%
Dermatologists (adult and pediatric)	95	25.3% [B]	21	15.1%	**.014**	116	22.5%
Vascular surgeons	40	10.6% [B]	7	5.0%	**.050**	47	9.1%

Census region	n	%	n	%		n	%
South	106	28.2%	54	38.8% [A]	**.021**	160	31.1%
Northeast	104	27.7%	29	20.9%	.118	133	25.8%
West	92	24.5%	28	20.1%	.295	120	23.3%
Midwest	74	19.7%	28	20.1%	.920	102	19.8%

Practice setting	n	%	n	%		n	%
Physician-owned private practice/clinic	203	54.0%	66	47.5%	.190	269	52.2%
Academic /research hospital or associated outpatient clinic	105	27.9%	37	26.6%	.770	142	27.6%
Non-academic (community) hospital or associated outpatient clinic	66	17.6%	33	23.7%	.119	99	19.2%
Government / VA hospital, other gov’t	1	0.3%	3	2.2%	*	4 [A]	0.8%
Specialized non-hospital-owned vascular clinic/center	1	0.3%	0	0.0%	*	1	0.2%

Member of a vascular anomaly team?	n	%	n	%		n	%
Yes	91	52.6% [B]	10	14.1%	** < 0.0001**	101	41.4%
No	82	47.4%	61	85.9% [A]	** < 0.0001**	143	58.6%

If part of a vascular anomaly team is this in a children’s hospital or unit?	n	%	n	%		n	%
Yes	63	69.2% [B]	4	40.0%	** < 0.0001**	67	66.3%
No	28	30.8%	6	60% [A]	** < 0.0001**	34	33.7%

Mean number of unique total patients, all conditions, treated in previous 12 months	n		n			n	
Mean	1168		1071		0.0634	1142	
Median	1200		1100			1200	
Standard deviation	506		574			526	

^*^Figures subject to rounding

^**^For each significant pair, the key (CAPITAL LETTERS A,B) of the smaller category appears within the category with the larger proportion. Significance level: .05

### Age distribution of cVM patients

Respondents reported that:

- 63% of patients were ≤ 18 years
- 37% were adults, including 11% ≥ 50 years

Estimated national cVM patients by age group:

-  < 12 months: 44,859
- 1–5 years: 29,518
- 6–18 years: 47,384
- 19–49 years: 50,879
-  ≥ 50 years: 21,556

**Fig. 3:**
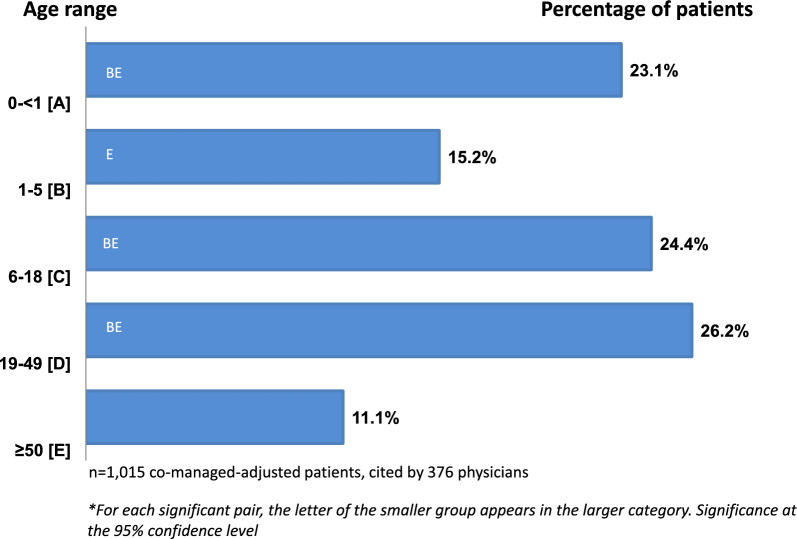
Patient age distribution. Estimated proportion of cVM patients by age group

Adults aged 19–49 represented the largest subgroup (26.2%) (*p* < 0.001) (Table 4, Fig. 3).

**Table 4:** Estimates of the number of annual cVM-treated patients by age

Estimated number of cVM patients by age^*,**^						
	A	B	C	D	E	
Age	0 < 12 months	1–5 years	6–18 years	19–49 years	≥ 50 years	Total
n =	44,859 [BE]	29,518 [E]	47,384 [ABE]	50,879 [ABCE]	21,556	194,195

^*^Weighted (co-managed adjusted) patients; based on proportion of study physicians’ patients by age group

^**^For each significant pair, the key (CAPITAL LETTERS A,B,C,D,E) of the smaller category appears within the category with the larger proportion. Significance level: .05

### Practice settings and comanagement

Of the 515 respondents:

- 52.2% practiced in physician-owned settings
- 47.8% were affiliated with hospital-based or academic centers
- 41.4% of hospital-based respondents belonged to a vascular anomaly team

Physicians affiliated with vascular centers saw significantly more cVM patients (3.9 vs. 1.1, p < 0.0001) than those outside such teams. Study physician responses, when calculated, indicated that 63.6% of cutaneous-only and 65.5% of mixed cases were comanaged, with vascular anomaly teams involved in 66% and 90% of these cases, respectively.

There were no statistically significant differences in the distribution by general practice setting between the 376 survey respondents who did and the 139 physicians who did not have cVM patients, but there were differences between those who were part of specialized vascular anomaly teams or in children’s units.

Physicians with cVM patients were significantly more likely to belong to vascular anomaly teams or malformation centers (*p* < 0.0001) than those without such patients, and of the physicians with cVM patients based at such centers, significantly more worked at children’s hospitals or units, compared with vascular-center-based physicians without target cVM patients (*p* < 0.0001) (Table 3).

## Discussion

This nationally representative study provides the first real-world estimate of the currently managed U.S. prevalence of cutaneous venous malformations (cVMs), addressing a critical gap in the vascular anomalies literature. We found that nearly 200,000 patients are treated annually by a multidisciplinary group of target specialists. While rare in absolute terms, cVMs represent a meaningful burden for the healthcare system and affected individuals, with clear implications for diagnosis, treatment, and research prioritization.

Our findings reinforce that cVM is a chronic, progressive disease affecting both pediatric and adult populations. Approximately 37% of patients were adults, highlighting the ongoing care needs beyond childhood. Yet, as previous reports by Iacobas and Blei have noted, specialty care options are often limited for adults, particularly outside dedicated vascular anomaly centers [18, 39]. This gap likely contributes to suboptimal disease control, fragmented care, and poor quality of life for many adult patients.

Importantly, 85% of surveyed physicians managing cVMs agreed that there is **a** significant unmet medical need**.** There are currently no FDA-approved pharmacologic therapies for cVM, and procedural interventions such as sclerotherapy or surgery often yield suboptimal results for superficial lesions. Given the known role of TEK and PIK3CA mutations in pathogenesis, emerging therapies targeting the PI3K/AKT/mTOR pathway—such as sirolimus—may offer promise, but their utility in dermatologically confined lesions remains to be fully established.

This study also demonstrates the central role of comanagement: More than 60% of cVM cases were managed by multiple specialists, often within interdisciplinary vascular teams. Vascular surgeons and dermatologists emerged as the top contributors to patient care, with vascular center–affiliated physicians reporting higher patient volumes. These findings highlight the need to support multidisciplinary infrastructure and to ensure that dermatologists are included in care pathways for vascular anomalies.

### Limitations

While our methodology allowed for robust population-level inferences, the study has several limitations. First, although the sample was stratified and weighted to reflect national demographics, certain specialist groups involved in cVM management (e.g., geneticists, otolaryngologists, plastic surgeons) were not surveyed due to feasibility constraints. Second, physician recall bias is inherent to survey-based methodologies. However, the rarity and clinical distinctiveness of cVM likely minimized such bias. Finally, the co-management adjustment model, while grounded in prior validated work, relied on fractional weights rather than direct patient-level linkage. Future research could refine this by integrating EMR-based patient registries or claims databases.

Despite these limitations, the study offers a foundational epidemiologic reference for cVM, with implications for clinical care, resource allocation, and drug development. As targeted therapeutics emerge, such real-world prevalence data are essential for health policy and regulatory planning, including orphan drug designation and trial feasibility.

## Conclusions

Our study is the first to estimate the national treatment prevalence of cutaneous venous malformations in the United States using a scientifically rigorous, blinded, and nationally representative methodology. We found that approximately 194,195 unique patients—including both children and adults—are treated annually by U.S. specialists.

Despite its rarity, cVM imposes a significant burden across the lifespan. The lack of FDA-approved therapies, limited access to adult care, and high rates of co-management underscore the pressing unmet need for safer and more effective treatments.

These findings highlight the urgency of advancing cVM-specific research, developing targeted pharmacologic interventions, and expanding access to comprehensive vascular anomaly care across all age groups.

## Data Availability

The datasets generated and/or analyzed during the current study are available from the corresponding author upon reasonable request.

## Abbreviations

US: United States
VM: Venous malformation
VA: Venous anomaly
cVM: Cutaneous venous malformation
FDA: Food and drug administration
LMC: Microcystic lymphatic malformations with a cutaneous component
AMA: American Medical Association
